# A Request

**Published:** 1847-06

**Authors:** 


ARTICLE IX.

*A Request.*

*Messrs. Editors:*—I wish to ask Dr. Henry, of Pekin, for a "Philosophical Theory" which shall sustain his practice of using opium in "fevers and inflammations." He has promised it if asked for through the Journal. It would add to the interest of the reply if a few cases were given of disease thus treated in which the effects of such doses were accurately noted. A SUBSCRIBER.

